# Orofacial manifestations of mucocutaneous leishmaniasis: a case series from Brazil

**DOI:** 10.12688/f1000research.19056.4

**Published:** 2020-09-24

**Authors:** Gleicy Gabriela Vitória Spinola Carneiro Falcão, Liliane Lins-Kusterer, Patricia Miranda Leite-Ribeiro, Viviane Almeida Sarmento

**Affiliations:** 1 Professor Edgard Santos Hospital, Federal University of Bahia, Salvador, BA, 40110060, Brazil

**Keywords:** Leishmaniasis, Mucocutaneous, Diagnosis, Oral, Dental Care

## Abstract

Dentists play a fundamental role in the early diagnosis of oral leishmaniasis. Although these lesions are rare at oral mucosa, this is one of the manifestations sites of the disease This study reports seven clinical cases of orofacial mucocutaneous leishmaniasis. All had leishmaniasis diagnosis confirmed by laboratory tests, with orofacial involvement. Five out of the seven cases were males, and in four cases, patients had associated comorbidities. Late diagnosis was observed, resulting in treatment delay and increased hospitalization stay. One patient had severe psychological consequences due to facial deformity. The lack of differential diagnosis due the great variability of clinical presentation of the lesions and frequent unspecific histopathology represent a challenge for the dentist. In two reported cases, there were unspecific biopsy results. This series of cases highlights the importance of a multidisciplinary approach in the diagnosis and treatment of oral and perioral leishmaniasis. Patients with atypical lesions, originating from or living in endemic regions, should be investigated for leishmaniasis. These procedures could avoid delays in diagnosis and decrease the risk of disease dissemination.

## Introduction

Leishmaniasis is a parasitic disease caused by several species of the protozoan genus *Leishmania* ^1^. It is a widely dispersed disease, being endemic in 98 countries, including Brazil. Leishmaniasis classification encompasses different clinical forms ^2^; mucocutaneous leishmaniasis is a chronic form of infection ^3^ that may manifest in the mucosa after months or years of latency ^4^.

The mucosal involvement of leishmaniasis is uncommon, mainly in immunocompetent individuals ^5^. The lymphatic or hematogenous dissemination of amastigotes may occur from the skin to the nasal, oropharyngeal, laryngeal and/or tracheal mucosa. Delayed diagnosis ^3, 6^ and development of primary lesion in the oral mucosa and in the head and neck region can cause dysphagia, dysphonia and dyspnoea ^3^.

The diagnosis of mucocutaneous leishmaniasis can be difficult ^7^. In older lesions, few parasites are usually detected by microscopy or culture and the clinical aspect may resemble neoplasia ^1, 8^. Orofacial symptoms depend on the localization of the lesions and may include nasal obstruction, difficulties in swallowing, mucosal bleeding and/or hoarseness ^8^. Destructive lesions of the mucosa contain few parasites, with high levels of tumor necrosis factor (TNF) suggesting an unmodulated immune response with increased production of proinflammatory cytokines responsible for tissue damage ^9^.

In this study we report seven clinical cases of orofacial mucocutaneous leishmaniasis from Brazil.

## Case reports

This study included seven patients admitted to Edgard Santos University Hospital, Federal University of Bahia, Brazil. All patients had a confirmed diagnosis of mucocutaneous leishmaniasis with oropharyngeal involvement and no visceral involvement, confirmed by laboratory tests. This study was approved by the Ethics and Research Committee of Edgard Santos University Hospital, CAAE 93381518.7.000.0049. All patients (or parents/guardians) provided written informed consent for the publication of their medical data and images.

### Case 1

**Figure 1.**
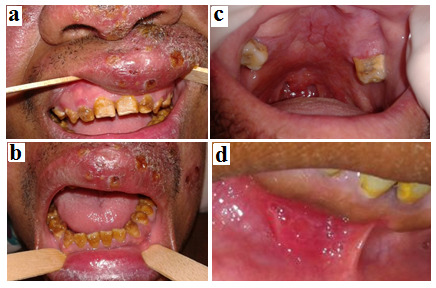
Oral examination evincing poor oral health status and amelogenesis imperfecta reported at Case-1 ( **a** and **b**); Infiltrative lesion on hard, soft palate and uvula reported at Case-2 ( **c**); Ulcerated lesions in the lower lip frenulum, reported at Case-3 ( **d**).

Male, 24-years-old, Caucasian, unemployed, from Tancredo Neves, State of Bahia, Brazil, was admitted to the University Hospital, in January 2012, presenting diffuse bullous lesions on the body, osteoarthritis of the distal interphalangeal joints and proteinuria 399 mg/day (reference value >150mg/day). He was diagnosed with systemic lupus erythematosus (SLE) and treated with mycophenolate mofetil (MMF). The starting dose for MMF was 0.5 g per day and it was increased up to 1 g per day intravenously. In 2014, two years after SLE diagnosis, he was hospitalized, presenting with ulcerated-painless-skin lesions on the face, upper lip, scalp, neck, upper and lower limbs. Oral examination evidenced crusty upper lip lesions, poor oral health status and amelogenesis imperfecta ( Figure 1a and 1b). He developed secondary infection associated with fever, and antibiotic therapy with cephalexin was initiated (1g/day) and a maintenance dose of prednisone (5 mg/day intravenously). On the third day, biopsies were performed on the left nasal mucosa and on the right lower limb lesions. The diagnosis of disseminated leishmaniasis was confirmed (positive PCR and Montenegro intradermal test). Liposomal amphotericin B was introduced on the fourth day of hospitalization at a dose of 150 mg/day up to a maximum dose of 2,400 mg. The patient treatment was followed-up for six months, and lesions were observed to have healed. One month later, during the follow-up for SLE, we observed new development of ulcerated skin lesions on the face and on the upper and lower right limbs. Blisters and fever were absent and the recurrence of disseminated leishmaniasis was confirmed. Few weeks later, the patient was admitted for treatment of new lesions, presenting with erythema, diffuse facial edema, lymphadenopathy and ulcerated and pustular lesions. Patient was treated with liposomal amphotericin B at a cumulative dose of 3,050 mg and followed-up until complete remission of the lesions. Currently, patient is under maintenance treatment for SLE.

### Case 2

In July 2013, 53-year-old male, Caucasian, unemployed, from Mundo Novo, State of Bahia, Brazil, attended to the Stomatology Clinic at University Hospital, presenting with pain, nasal obstruction, and complaints of odynophagia and dysphagia. Physical examination showed painful, hyperemic and friable lesion in the right nasal cavity, associated with infiltrative lesion on the hard and soft palate, and uvula ( Figure 1c). We observed ulcerated lesion on the left eyebrow and right eye with seropurulent secretion, a small ulcer on the lower eyelid, on the lobe of the right ear and a lesion in the malar region. The patient was admitted for diagnosis and treatment of disseminated skin lesions. A biopsy of the palate lesions revealed a non-specific erosive chronic inflammatory process. The patient was HIV negative and positive for Montenegro reaction. Treatment with amphotericin B was initiated at a dose of 150 mg/d up to a maximum dose of 2,410 mg. Lesions regressed after drug treatment and oral treatment was initiated during hospitalization. We removed dental foci without any intercurrence. One month later, the patient was discharged. However, in August 2013, in outpatient medical consultation, the lesions were observed in nasal mucosa and palate. He was followed up in the outpatient clinic and treatment with glucantime 20 mg/kg/day was prescribed for one month. The follow-up period was eight months, and the result was negative.

### Case 3

Female, 31 years old, Caucasian, unemployed, from Salvador, State of Bahia, was diagnosed (Montenegro positive test) with American Tegumentary Leishmaniasis in October, 2011. The patient was treated with Glucantime, 20 mg/kg/day for 30 days. A lesion in her back region was partially healed. In 2012, two episodes of recurrence occurred and restarted treatment with Glucantime in January and May. In a third recurrence episode (August, 2012), due to the maintenance of the lesion, a lesion biopsy was performed and *Leishmania braziliensis* was diagnosed. Treatment with amphotericin B was initiated at a dose of 250 mg/d up to a maximum dose of 2,400 mg, resulting in wound healing. In 2013, the patient was admitted with submandibular lymphadenopathy and ulcerated lesions in the lower lip frenulum ( Figure 1d), gingiva, nasal septum and in the back region. She was hospitalized for diagnosis and treatment of lesions with liposomal amphotericin B. Due to persistence of the lesions, HIV serology was performed. The patient was HIV positive and antiretroviral therapy was started (efavirenz 600mg, tenofovir 300mg, lamivudine 300mg, per day, one tablet containing the three drugs). Excisional biopsies of oral lesions were performed with unspecific result. Microbiological analysis for fungi was negative. Two months later, the patient was discharged and a maintenance dose of liposomal amphotericin B (150 mg/day) was prescribed.

### Case 4

**Figure 2.**
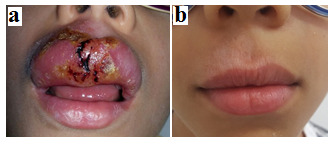
Hyperemic and pruritic lesion on the upper lip, reported at Case-4 ( **a**); Aspect of the upper lip one month later, evidenced remission of lesion ( **b**).

In 2017, an eight year-old Caucasian male from Salvador, Bahia, Brazil, presented with a hyperemic and pruritic lesion on the upper lip which had persisted for six months. Patient was treated with acyclovir cream, 5%, 5 times/day and cefadroxil (50 mg/kg/day) for seven days, with no response. He presented worsening of the lesion and Montenegro intradermal examination was performed ( Figure 2a). The patient was positive for American Tegumentary Leishmaniasis. Treatment with glucantime (10 mg/day) for 20 days was initiated. After three days of treatment, the patient developed vomiting episodes, intermittent fever, diarrhea, hypoglycemia, dark urine, and began developing a reaction of cardiotoxicity and hepatoxicity. Treatment with liposomal amphotericin B was initiated (3 mg/kg/day for 5 days, followed by 3 mg/kg). One month later, patient was discharged with remission of the lesion ( Figure 2b). Two months later, the patient was admitted at University Hospital with a new, erythematous and ulcerated lesion on the upper lip lesion, lymphadenopathy, and facial edema. Therapy with amoxicillin 250 mg (1g/day) and amphotericin B (100 mg/day) for 10 days was started. Patient is currently in psychological follow-up due to trauma caused by facial disfiguration and difficulty in returning to social life. Patient maintained outpatient follow-up and did not present with recurrence of the lesion.

### Case 5

**Figure 3.**
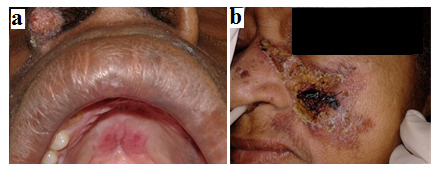
Ulcerated oral lesions in hard palate reported at Case-5 ( **a**). Leishmaniasis lesion in the left malar region reported at Case-6 ( **b**).

In 2008, a male, 30 years old, Caucasian, unemployed, HIV – negative, with no other concomitant infections, presented with an isolated nodulation in the right leg and he was diagnosed with Tegumentary leishmaniasis. The patient was treated with Glucantime (10 mg/kg/day for 20 days), achieving complete healing of the lesions. In 2014 the patient presented a papule in the inferior eyelid of the right eye. Patient was PCR positive for *Leishmania brasiliensis*. Lesions progressively appeared in different body surfaces such as the chest, abdomen, back, feet, and mouth. Ulcerated oral lesions were present in the hard palate, as well as the left and right jugal mucosa ( Figure 3a).

Progression of disease was associate with fever, headache and weight loss. Treatment with glucantime (20 mg/day) for 30 days followed by treatment with amphotericin B at a cumulative dose of 1.5 to 2 g, 50 mg/day. Patient developed acute renal failure secondary to the use of amphotericin B. Treatment was replaced by the liposomal form at a dose of 100 mg/day and patient was discharge one month later with complete remission of lesions.

### Case 6

Female, 59 years old, Caucasian, unemployed, with diabetes, hypertension, congestive heart failure, chronic renal disease and paraparesis secondary to Human T-cell leukemia virus type 1 (HTLV-1) infection. In June 2012, patient presented with a papule lesion in the left malar region with late ulceration and increasing in size ( Figure 3b). After 15 days, another lesion developed in the right knee. Patient was positive for Montenegro intradermal test and diagnosed with mucocutaneous leishmaniasis and was admitted in the University Hospital in September 2012. Patient developed hyperkalemia and, after stabilization of renal function, treatment with liposomal amphotericin B (100 mg/day) was introduced. One day after, the patient developed another episode of renal dysfunction and therapy was discontinued. Five days later, therapy was reintroduced, alternating with dialysis. The culture examination of the malleolar lesion was performed, being positive for *Proteus vulgaris* and hemoculture was positive for *Staphylococcus aureus*. In October 2012, patient was transferred to intensive care unit and developed multiple organ failure, dying two weeks later.

### Case 7

**Figure 4.**
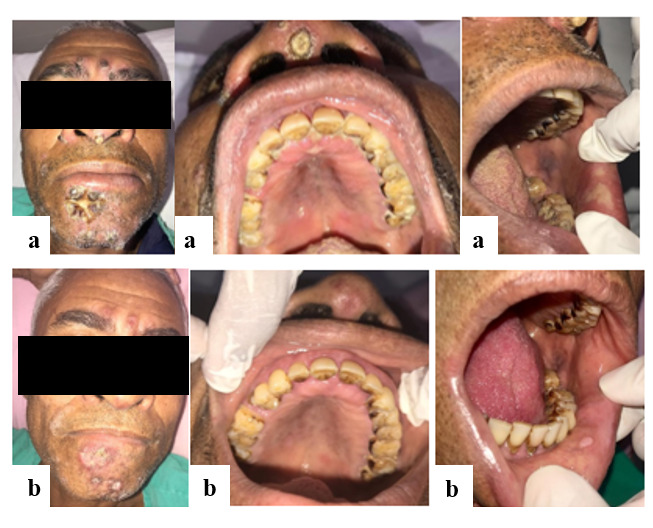
Ulcerated lesions in face, marginal gingiva, palate, and labial mucosa, before ( **a**) and after treatment with liposomal amphotericin B ( **b**) reported at Case-7.

Male, 59 years old, mixed ethnicity, unemployed, previously healthy, reported the appearance of an erythematous-crusty lesions in the mental protuberance region, evolving in two months to other parts of the body such as frontal and occipital regions, nasal septum, ears, hands, and lower limbs. Oral cavity clinic-examination showed scattered ulcers on the face, lower labial mucosa, and on the left lip commissure, pseudomembrane on the marginal gingiva, and an exophytic nodule in the left labial mucosa ( Figure 4). Patient was Montenegro intradermal test positive and was admitted at the University Hospital in December 2018. The PCR analysis was perfomed in a laboratory outside the hospital. After admission, we observed enlarged lymph nodes of hard consistency in the left inguinal region, and an extensive melanocytic lesion in the left plantar region. The lesion was irregular, presenting an area of hyperkeratosis with a grey-bluish center. The patient was biopsied and the diagnostic hypothesis of melanoma was confirmed. We requested laboratory and imaging tests for melanoma staging. After seven days, we accessed the PCR laboratory test and initiated therapy with intravenous liposomal amphotericin B 50 mg at the dose of 200 mg/kg/day for 15 days ( Figure 4). Diagnostic confirmation of melanoma resulted in the excision of the melanocytic lesion with left inguinal lymphadenectomy. Patient was referred to an oncology center. The patient has not yet returned for evaluation as they are receiving antineoplastic treatment outside our hospital.

## Discussion

Five out of the seven cases were males from Brazil’s endemic regions. Four cases had associated comorbidities (SLE, HIV, HTLV infection and melanoma). A multicenter case series study ^1^ with seven patients presenting oral leishmaniasis reported higher frequency of oral lesions in males (86%), tongue (57%) with predominance of exophytic lesions (85%). In our case series, patients were predominantly males with ulcerated lesions in the lips.

The Montenegro reaction is a diagnosis test of high sensitivity, low cost and minimally invasive. Serological tests, such as immunoenzymatic assays and indirect immunofluorescence, show variation in their results depending on the applied technique and disease classification ^10^. In our series of cases, late diagnosis was observed resulting in treatment delay and extension of hospitalization stay.

Facial involvement of leishmaniasis is a serious complication, since it can lead to disfiguration and be potentially fatal ^11, 12^. In one case reported, the patient had severe psychological consequences due to facial deformity, reinforcing the importance of early diagnosis and appropriate therapy.

Unusual manifestations as DisL or purely oral Leishmaniasis should be considered in immunocompromised patients ^13– 16^. In immunocompetent patients, primary and exclusive mucosal involvement in the head and neck region is uncommon; lesions affecting the buccal mucosa exclusively are even rarer ^1, 15– 19^. In our series of cases, four cases (57.1%) presented some level of immunological deficiency.

Leishmaniasis is difficult to treat, and may present with spontaneous reactivation ^20^ or be transmitted by a transplanted organ ^21^. Control of cutaneous leishmaniasis depends on case management, early detection and appropriate treatment ^22^. We observed cases of adverse drug reactions during treatment and protocol changes were necessary during the course of treatment. We also observed frequent recurrence of lesions, probably associated with immunossupression and therapeutic failure with inadequate treatment suspension or suboptimal doses.

Oral lesions of leishmaniasis are rare; however, oral mucosa may be one of the manifestations sites of the disease. In this context, the dental surgeon plays a fundamental role in the early diagnosis of oral lesions of leishmaniasis ^3^. The great variability of clinical presentation of the lesions and frequent unspecific histopathology represent a challenge in regard to differential diagnoses. The dental surgeon can contribute to early diagnosis of mucosal lesions, since oral mucosa may be the primary site of the disease manifestation.

Although histopathological techniques describe the inflammatory infiltrate associated to leishmaniasis, they present low diagnostic specificity. The granulomatous aspect of lesions in later stages of cutaneous infection of leishmaniasis hampers histopathological analysis, since few parasites can be found in these lesions ^1, 7, 23^. In our reported cases, we had two unspecific biopsy results.

Differential diagnosis of mucosal lesions should include mucosal leishmaniasis. The variation in the clinical presentation of leishmaniasis and its ability to mimic different diseases represent a challenge for disease diagnosis. Due to the granulomatous ulcerated aspect of leishmaniasis lesions, squamous cell carcinoma and deep fungal infections, such as paracoccidioidomycosis and histoplasmosis, are differential diagnoses. In the reported cases, negative biopsies and cultures for these mycoses, and the absence of malignant neoplasia in histological sections, followed by the cure of lesions treated with amphotericin, in patients with confirmed skin leishmaniasis lesions by Montenegro reaction or PCR, confirmed that the all described lesions were oral manifestations of leishmaniasis.

There is no specific standardization for mucocutaneous leishmaniasis therapy ^17, 22^. The cases we reported were submitted to different therapeutic plans, adjusted to each patient. In our case series, all patients received systemic treatment for mucocutaneous leishmaniasis, because of this disease well-known resistance. Alternative topical treatment includes use of ointment, cryotherapy, and intralesional injection with antimonials. Multiple and large lesions compromising the face are less suitable for local therapy. The treatment of mucosal leishmaniasis are still based on case reports ^9^.

In our cases, all patients were treated with systemic medication. We presented a case with primary and exclusive lesion on the lip. Local treatment was not administered, and the patient is under follow-up. 

Our findings present some limitation. First, the few cases reported are not a representative population sample, limiting any possible inference. Due to socioeconomic reasons, patients living far from Salvador are not accessible for a close follow-up and dental care. Diagnosis based on oral biopsies are very limited and the dental surgeon must be aware of the diverse clinical forms of leishmaniasis. Cases of orofacial mucosa leishmaniasis are rare, but we should be aware of them during oral examination. We agree that our report may contribute to a better dental evaluation and early diagnosis of cases of oral leishmaniasis. 

## Conclusions

The present study highlights the importance of a multidisciplinary approach in the diagnosis and treatment of orofacial leishmaniasis. Patients that travelled or live in endemic regions and presenting atypical lesions should be investigated for leishmaniasis. This could avoid delays in diagnosis and decrease the risk of the disease dissemination.

## Data availability

### Underlying data

All data underlying the results are available as part of the article and no additional source data are required.
