# Supplementary material for: Orofacial manifestations of mucocutaneous leishmaniasis: a case series from Brazil
Source: F1000Res. 2020 Sep 24;8:756. Originally published 2019 May 29. [Version 4] doi: 10.12688/f1000research.19056.4 (PMC7527865; doi:10.12688/f1000research.19056.4)
Supplement: Peer review history

# Peer review: Reviewer response for version 4

**DOI:** 10.5256/f1000research.29234.r71901

**Published:** 2020-09-30

**Authors:** Braulio Valencia

**Affiliations:** 1 Viral Immunology Systems Program (VISP), Kirby Institute, The University of New South Wales, Kensington, Australia

Nothing to comment.

Are enough details provided of any physical examination and diagnostic tests, treatment given and outcomes?

Partly

Is the background of the cases’ history and progression described in sufficient detail?

Partly

Is sufficient discussion included of the importance of the findings and their relevance to future understanding of disease processes, diagnosis or treatment?

Partly

Is the conclusion balanced and justified on the basis of the findings?

Partly

Reviewer Expertise:

Infectious and Tropical Diseases, Neglected Tropical Diseases, New-World Leishmaniases.

I confirm that I have read this submission and believe that I have an appropriate level of expertise to confirm that it is of an acceptable scientific standard.

# Peer review: Reviewer response for version 3

**DOI:** 10.5256/f1000research.26813.r65109

**Published:** 2020-06-22

**Authors:** Braulio Valencia

**Affiliations:** 1 Viral Immunology Systems Program (VISP), Kirby Institute, The University of New South Wales, Kensington, Australia

Observation case 6: it's up to the editors consider if this case is suitable to be incorporated or not in the field of dentistry or oral health. In the lack of oral involvement, there is no rationale to include this case.

 Observation case 7: if a confirmatory PCR was performed, authors must clearly state this PCR was based on a sampling obtained from the oral lesion. Due to this is a DisL case, PCR sample could be obtained from any of the more accessible samples.

 Observation paragraph 4: the sentence "Leishmaniasis should be considered in immunocompromised patients" is unclear (it is assuming any Leishmaniasis, even localised cutaneous Leishmaniasis should be ....).  "Unusual manifestations as DisL or purely oral Leishmaniasis should be considered in immunocompromised patients" is probably more accurate and supported by their findings.

 Observation regarding therapy: again, references 17 and 21 are case reports. The authors must familiarise with evidence-based recommendations as the one provided in the prior review. Local treatment is exclusively recommended to localised cutaneous leishmaniasis. It's a wrong and dangerous recommendation to suggest local therapies are suitable for the treatment of mucosal leishmaniasis.

Are enough details provided of any physical examination and diagnostic tests, treatment given and outcomes?

Partly

Is the background of the cases’ history and progression described in sufficient detail?

Partly

Is sufficient discussion included of the importance of the findings and their relevance to future understanding of disease processes, diagnosis or treatment?

Partly

Is the conclusion balanced and justified on the basis of the findings?

Partly

Reviewer Expertise:

Infectious and Tropical Diseases, Neglected Tropical Diseases, New-World Leishmaniases.

I confirm that I have read this submission and believe that I have an appropriate level of expertise to confirm that it is of an acceptable scientific standard, however I have significant reservations, as outlined above.

# Response

**Published:** 2020-08-30

**Authors:** Viviane Sarmento

**Affiliations:** Federal University of Bahia, Brazil

Dear Reviewer,

 We appreciate your comments and made some changes to the manuscript. In case 7, the PCR analysis was performed in a laboratory outside the hospital. Unfortunately, it was not possible to identify exactly where the sample was collected for PCR analysis. We added this information.

 In relation to paragraph 4, we have changed the phrase “Leishmaniasis should be considered in immunocompromised patients” by the phrase “Unusual manifestations as DisL or purely oral leishmaniasis should be considered in immunocompromised patients”.

 Regarding therapeutics, we chose to exclude the references and the paragraph from the discussion about the controversial findings in the literature regarding the local treatment of leishmaniasis.

 Thank you very much.

 The authors

# Peer review: Reviewer response for version 2

**DOI:** 10.5256/f1000research.25383.r62518

**Published:** 2020-05-11

**Authors:** Elismauro Francisco Mendonça

**Affiliations:** 1 Department of Oral Medicine (Oral Pathology), Dental School, Federal University of Goiás, Goiânia, Brazil

This paper is very interesting. It is well written and with scientific evidences. This lesion has clinical diagnosis very complex and the criteria used by the authors was well discussed in the paper. 

**Are enough details provided of any physical examination and diagnostic tests, treatment given and outcomes?**

 Yes, the authors have showed for each case presented the investigation methods including diagnostic tests, as well as, the treatment. Although in some cases there was recurrence the lesion after protocol treatment, we believe that economic condition of the patients might contribute to unsuccessful treatment. Other point is the access to public health system in the country nor always favorable. In addition, the authors have discussed this aspect and limitations.

**Is sufficient discussion included of the importance of the findings and their relevance to future understanding of disease processes, diagnosis or treatment?**

 The discussion section was well presented , however would be interesting the authors to include which is the best scientific evidence for treatment in the actual moment? For example, glucantinme or anphotericin ? Or other?

**Is the case presented with sufficient detail to be useful for other practitioners?**

 Yes, but in my opinion in the discussion section the authors should emphasize the high index of recurrence associated to treatment protocol used and comorbidities. Maybe the protocol treatment was not followed by the patient as prescribed by medical team.

**Is the background of the case’s history and progression described in sufficient detail?**

 Yes, the cases are well presented and clinical history can be easily followed by the reader.

 ----------------------------------------------------------------------------------------------------------------------------------------------------------

Are enough details provided of any physical examination and diagnostic tests, treatment given and outcomes?

Yes

Is the background of the cases’ history and progression described in sufficient detail?

Yes

Is sufficient discussion included of the importance of the findings and their relevance to future understanding of disease processes, diagnosis or treatment?

Yes

Is the conclusion balanced and justified on the basis of the findings?

Yes

Reviewer Expertise:

NA

I confirm that I have read this submission and believe that I have an appropriate level of expertise to confirm that it is of an acceptable scientific standard.

# Peer review: Reviewer response for version 2

**DOI:** 10.5256/f1000research.25383.r62403

**Published:** 2020-04-21

**Authors:** Braulio Valencia

**Affiliations:** 1 Viral Immunology Systems Program (VISP), Kirby Institute, The University of New South Wales, Kensington, Australia

The following observations have not been addressed:

1. Case 6: This is purely a DisL case. Due to the lack of oral involvement, this case is eminently of the dermatology or infectious diseases fields.
2. Case 7: as in case 6, no substantial evidence was provided to assume oral involvement. Authors described "we accessed the PCR laboratory test", but it's not clear where was this sample obtained. Partial improvement of the oral lesion after AMB is not conclusive to assume the parasitic etiology.
3. Paragraph 4: there is no reasonable evidence suggesting MCL is generally associated with VL or immunosuppression. All references supporting this statement (11-19) are case reports informing unusual clinical presentations. It's highly recommended to authors review https://doi.org/10.1016/B978-0-323-55512-8.00104-6 for a better understanding of the pathobiology and risk factors of MCL.
4. Paragraph 8 and 9: authors must review the therapeutic section of https://doi.org/10.1016/B978-0-323-55512-8.00104-6 to understand how differently pure mucosal lesions and cutaneous lesions are treated. In the context of oral leishmaniasis, the suggestion of topical therapy for mucosal lesions is unacceptable.

These are significant imprecisions which, if not addressed, are going to generate a deficient review.

Are enough details provided of any physical examination and diagnostic tests, treatment given and outcomes?

Partly

Is the background of the cases’ history and progression described in sufficient detail?

Partly

Is sufficient discussion included of the importance of the findings and their relevance to future understanding of disease processes, diagnosis or treatment?

Partly

Is the conclusion balanced and justified on the basis of the findings?

Partly

Reviewer Expertise:

Infectious and Tropical Diseases, Neglected Tropical Diseases, New-World Leishmaniases.

I confirm that I have read this submission and believe that I have an appropriate level of expertise to confirm that it is of an acceptable scientific standard, however I have significant reservations, as outlined above.

# Response

**Published:** 2020-05-20

**Authors:** Viviane Sarmento

**Affiliations:** Federal University of Bahia, Brazil

Dear reviewer,

Thank you for your considerations about the paper entitled “Orofacial manifestations of mucocutaneous leishmaniasis: a case series from Brazil”. Each point will be discussed below:

  

Case 6: This is purely a DisL case. Due to the lack of oral involvement, this case is eminently of the dermatology or infectious diseases fields.

We appreciate your considerations. However, we would like to highlight that we have proposed to report, not only cases with oral involvement, but also mucocutaneous leishmaniasis on the face, because the dentist also evaluates this area. Therefore, case 6 fits the pre-established criteria.

2. Case 7: as in case 6, no substantial evidence was provided to assume oral involvement. Authors described "we accessed the PCR laboratory test", but it's not clear where was this sample obtained. Partial improvement of the oral lesion after AMB is not conclusive to assume the parasitic etiology.

Thank you for your comments. Case 7 was first diagnosed with mucocutaneous leishmaniasis by the Monte Negro test, performed in the patient's city of origin. The patient was referred to the hospital for treatment. However, the treatment with amphotericin was only initiated after PCR confirmation.

3. Paragraph 4: there is no reasonable evidence suggesting MCL is generally associated with VL or immunosuppression. All references supporting this statement (11-19) are case reports informing unusual clinical presentations. It's highly recommended to authors review https://doi.org/10.1016/B978-0-323-55512-8.00104-6 for a better understanding of the pathobiology and risk factors of MCL.

There are reports of cases of leishmaniasis with the involvement of the oral mucosa in patients with immunosuppression (HIV, transplant patients), but this does not associate the disease with such risk factors. We decided to change in the text to the following sentence: Leishmaniasis should be considered in immunocompromised patients.

4. Paragraph 8 and 9: authors must review the therapeutic section of https://doi.org/10.1016/B978-0-323-55512-8.00104-6 to understand how differently pure mucosal lesions and cutaneous lesions are treated. In the context of oral leishmaniasis, the suggestion of topical therapy for mucosal lesions is unacceptable.

Thanks for your comments. However, according to the cited reference concerning local therapy, the "experience with local therapy for New World CL is limited, as local therapy was long considered unsuitable, especially in those patients infected with L. (V.) Braziliensis or L. (V. ) panamensis because of the potential risk of ML and the expectation that systemic treatment could prevent metastasis and mucosal involvement. However, systemic treatment does not guarantee the prevention of later ML. World CL."

# Peer review: Reviewer response for version 1

**DOI:** 10.5256/f1000research.20884.r55589

**Published:** 2019-11-04

**Authors:** Braulio Valencia

**Affiliations:** 1 Viral Immunology Systems Program (VISP), Kirby Institute, The University of New South Wales, Kensington, Australia

The scope of this article is to provide essential clues about orofacial manifestations of mucocutaneous leishmaniasis. Considering the target readers are Dental Surgeons, this manuscript has several imprecision's and inaccurate messages which need to be corrected.

**Abstract section:**

- The affirmation that "oral mucosa may be the primary site of the disease manifestation" is contradictory with the discussion and findings of this report as well as the existent and well-characterised clinical description of MCL. Almost all cases (except case 4) were manifested as disseminated Leishmaniasis with a significant and predominant cutaneous involvement. In the discussion section, paragraph four, line 5, authors state that primary and exclusive oral mucosa involvement is exceptionally uncommon. Then, what is described in the abstract is contradictory.
- Regarding that "All had mucocutaneous leishmaniasis with oropharyngeal involvement" is also incorrect. Due to the lack of detail in cases 5-7, it is not possible to accept these cases are definitively MCL. Case 6 is not a case of MCL; then, it needs to be removed from this article.

**Introduction section:**

- Paragraph 2, line 1-2: It's not clear if authors suggest that MCL is particularly frequent in immunosuppressed individuals. This affirmation is highly dependent on the prevalence of immunosuppressive comorbidities (as HIV). Considering that immunosuppressive conditions are not highly prevalent in endemic areas (except some African or Asian regions), this affirmation is inaccurate. Mucosal involvement in new-world Leishmaniases ranges from 5-20% ^1^. Then, in any case, this affirmation is not correct or requires clarification.
- Paragraph 2, line 5-6: development of primary lesions in oral mucosa is very infrequent and mainly described in old-world Leishmaniasis ^2^ OR in individuals with immunosuppressive conditions (4/7 cases in this report). For this reason, authors must consider changing the scope (title as well?) of this review from orofacial manifestations of MCL to atypical manifestations of leishmaniasis among immunosuppressed individuals.

**Case reports:**

- Case 1: more than MCL, this is a case of disseminated leishmaniasis (DisL) in an immunosuppressed individual. Here the predominant mucosal involvement appears to be nasal (no details are provided regarding the degree of nasal involvement), and the oral involvement is confined to the upper lip involvement. This is unusual even in immunosuppressed individuals, considering that lips, gums, tongue, and hard palate are extremely infrequent in new-world MCL ^3^.
- Case 2: again, this is a case of DisL in an immunocompetent individual. Here the mucosal involvement is more typical, but an HIV seronegative status is not enough to classify the patient as immunocompetent. A better characterisation of this individual is required.
- Case 3: again, another case of DisL in an immunocompromised patient. It's recommended to improve the quality of picture 1d. As mentioned before gums are unusual in new-world MCL. MCL is not commonly associated with lymphadenopathy. Both findings, and in the absence of parasitologic or histologic characterisation of the oral lesion makes it essential to consider other infectious diseases, importantly in an HIV-seropositive individual.
- Case 4: this case is MCL, but the pathophysiology is different from the prior cases. Here, more than a lymphatic/hematologic dissemination, what generated the MCL was a direct inoculation on the lip or a skin inoculation close to a mucosal structure. This case is clearly, utterly different from the rest and hard to classify as an unusual manifestation or among immunosuppressed individuals.
- Case 5: again, another DisL in an apparently immunocompetent patient. No details were provided here regarding the HIV serologic status or other potential sources of immunosuppression.
- Case 6: again, another DisL in an immunosuppressed individual. In this case, there is not any oral or mucosal involvement (only cutaneous lesions are described). Then, this case must be removed from the report.
- Case 7: there is a lack of evidence to catalogue this case as MCL. Besides the facial cutaneous lesions (only localised cutaneous leishmaniasis?), lesions described in the oral mucosa are unusually located, and an alternative explanation must be considered (metastatic melanoma?). It's not clear where the sample for PCR was obtained. With this unclear clinical and parasitological description, it's inaccurate to define the case as MCL.

**Discussion:**

- Paragraph 3, lines 1-2: MCL is potentially fatal, mainly when larynx or trachea are affected. From that scenario, it's incorrect to describe them as a facial involvement.
- Paragraph 4, lines 1-2: as suggested in the first observation of the introduction section, authors are suggesting MCL is generally associated with VL or immunosuppression. That is probably acceptable in the context of Sudanese MCL; however, this is not the epidemiological context of the study. Regarding immunosuppression, as discussed in the same observation, due to the lack of coexistence between Leishmaniasis and immunosuppressive conditions, there is not any evidence supporting this affirmation. The references used to support this statement are case reports which were not designed to measure the prevalence of MCL among immunosuppressed individuals.
- Paragraph 5, lines 6-7: the high rate of recurrences observed in these cases are not necessarily related to therapeutic issues. Immunosuppression is probably the primary determinant of therapeutic failure.
- Paragraph 6, lines 6-8: again, this affirmation is contradictory with the findings of this report. Only one case was purely a "primary" MCL, in the rest, the predominant manifestation was the development of disseminated cutaneous lesions.
- Paragraph 8, lines 5-6: the reason why systemic therapy was administered in all these cases is that systemic treatment is the only standard therapeutic regimen for MCL (not due to resistance issues). For a better understanding of current therapeutic recommendations, authors must review citation four ^4^ and update reference 9.
- Paragraph 9: no local therapy is currently recommended for MCL. This information must be improved or removed.

**Conclusions:**

- Paragraph 2: what other infections must be considered as a differential diagnosis among oral lesions?

Are enough details provided of any physical examination and diagnostic tests, treatment given and outcomes?

Partly

Is the background of the cases’ history and progression described in sufficient detail?

Partly

Is sufficient discussion included of the importance of the findings and their relevance to future understanding of disease processes, diagnosis or treatment?

Partly

Is the conclusion balanced and justified on the basis of the findings?

Partly

Reviewer Expertise:

Infectious and Tropical Diseases, Neglected Tropical Diseases, New-World Leishmaniases.

I confirm that I have read this submission and believe that I have an appropriate level of expertise to state that I do not consider it to be of an acceptable scientific standard, for reasons outlined above.

## References

[1] : Leishmaniases in Bolivia: Comprehensive Review and Current Status. *The American Journal of Tropical Medicine and Hygiene*.2009;80(5) : 10.4269/ajtmh.2009.80.704 704-711 10.4269/ajtmh.2009.80.704 19407110

[2] : 2. Mucosal leishmaniasis. *Transactions of the Royal Society of Tropical Medicine and Hygiene*.2001;95: 10.1016/S0035-9203(01)90217-2 S19-S26 10.1016/S0035-9203(01)90217-2 11370249

[3] : Diagnosis and Treatment of Leishmaniasis: Clinical Practice Guidelines by the Infectious Diseases Society of America (IDSA) and the American Society of Tropical Medicine and Hygiene (ASTMH). *Am J Trop Med Hyg*.2017;96(1) : 10.4269/ajtmh.16-84256 24-45 10.4269/ajtmh.16-84256 27927991PMC5239701

[4] : Leishmaniasis.2013; 10.1016/B978-1-4160-4390-4.00099-0 739-760 10.1016/B978-1-4160-4390-4.00099-0
